# Supplementary material for: Serotonergic neuron-glioma interactions drive high-grade glioma pathophysiology
Source: bioRxiv. 2025 Dec 12:2025.12.10.693579. Preprint. [Version 1] doi: 10.64898/2025.12.10.693579 (PMC12715541; doi:10.64898/2025.12.10.693579)
Supplement: Supplement 1 [file NIHPP2025.12.10.693579v1-supplement-1.pdf]

bioRxiv preprint doi: https://doi.org/10.64898/2025.12.10.693579; this version posted December 12, 2025. The copyright holder for this preprint (which was not certified by peer review) is the author/funder, who has granted bioRxiv a license to display the preprint in perpetuity. It is made available under aCC-BY 4.0 International license.

Supplementary Figure S1

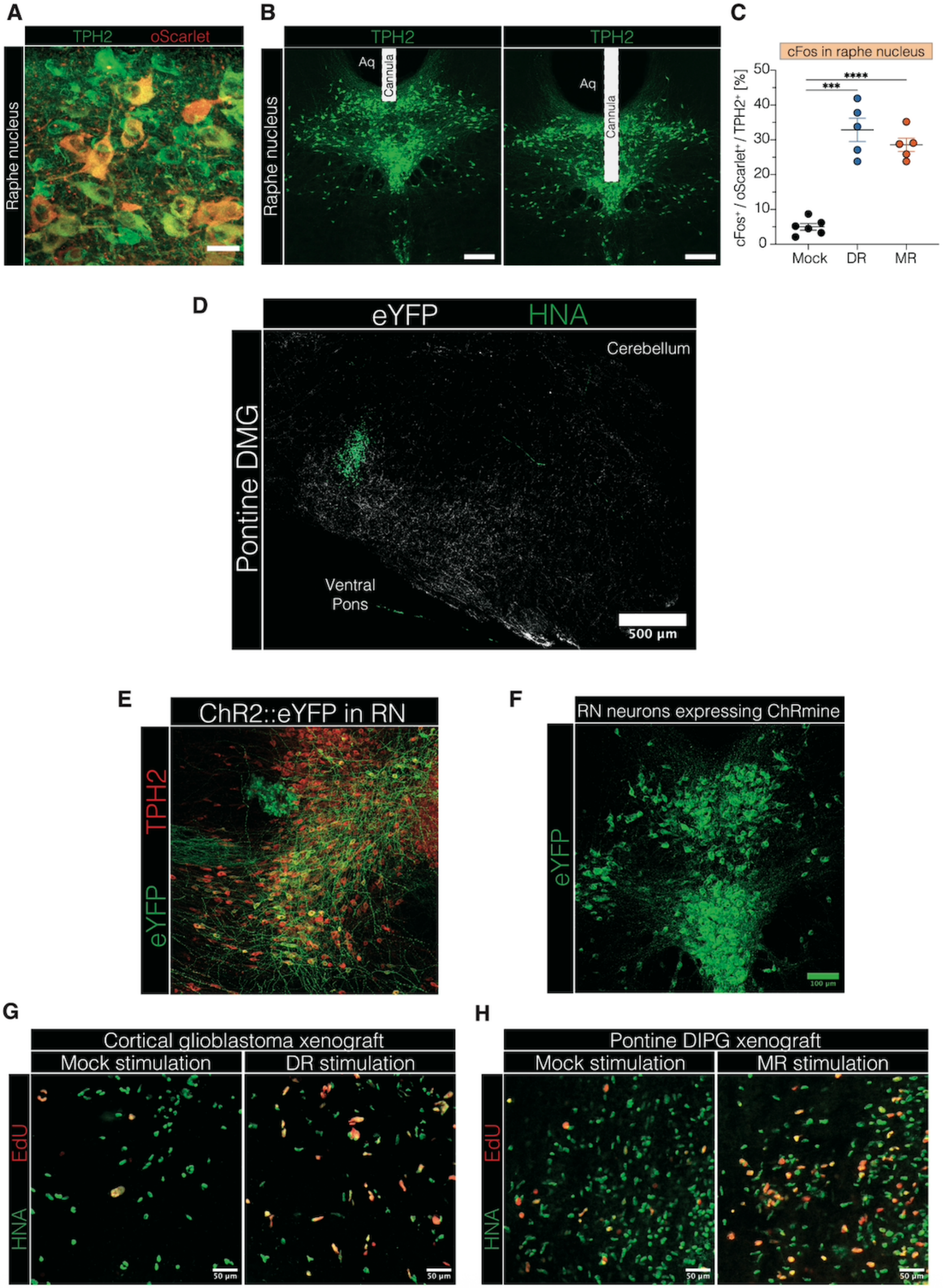

bioRxiv preprint doi: https://doi.org/10.64898/2025.12.10.693579; this version posted December 12, 2025. The copyright holder for this preprint (which was not certified by peer review) is the author/funder, who has granted bioRxiv a license to display the preprint in perpetuity. It is made available under aCC-BY 4.0 International license.

Fig. S1: 5HT neuron expression within the raphe nucleus and activity-mediated glioma proliferation effects.

- **A.** Representative confocal image showing oScarlet expression in the raphe nucleus of SERT-Cre x Ai230 mice. oScarlet: red, TPH2: green, scale bar = 20 µm.
- **B.** Confocal micrographs showing representative fiberoptic cannula placement for optogenetic experiments in the DR (left) and MR (right). TPH2: green, scale bar = 200 µm.
- **C.** Quantification of cFos expression following optogenetic stimulation of 5HT neurons in the DR or MR compared to mock-stimulated controls in SERT-Cre x Ai230 mice during a single stimulation session (t = 30 minutes) ('Mock', 'DR', and 'MR', n = 5 mice). One-way ANOVA with Tukey's post hoc test; ****$p < 0.0001$, ***$p < 0.001$. Data are presented as mean ± SEM.
- **D.** Representative confocal image showing virally labeled 5HT neurons within the ventral pontine glioma microenvironment in SERT-Cre mice. eYFP: red, HNA: green, scale bar = 500 µm.
- **E.** Representative confocal micrograph showing viral labeling (AAV-DJ-EF1α-DIO-hChR2(H134R)::eYFP) of MR^{5HT} neurons in a sagittal section. eYFP: green, TPH2: red, DAPI: white.
- **F.** Representative confocal micrograph showing viral labeling (rAAVPHP.eB-Tph2::ChRmine-eYFP) of RN^{5HT} neurons. eYFP: green, scale bar = 100µm.
- **G.** Confocal micrographs showing proliferating HNA^+ glioblastoma cells in M2 cortex in mock-stimulated (left image) and DR-stimulated (right image) mice. HNA: green, EdU: red, scale bars = 50µm.
- **H.** Confocal micrographs showing proliferating HNA^+ DMG cells in ventral pons in mock-stimulated (left image) and MR-stimulated (right image) mice. HNA: green, EdU: red, scale bars = 50µm.

bioRxiv preprint doi: https://doi.org/10.64898/2025.12.10.693579; this version posted December 12, 2025. The copyright holder for this preprint (which was not certified by peer review) is the author/funder, who has granted bioRxiv a license to display the preprint in perpetuity. It is made available under aCC-BY 4.0 International license.

Supplementary Figure S2

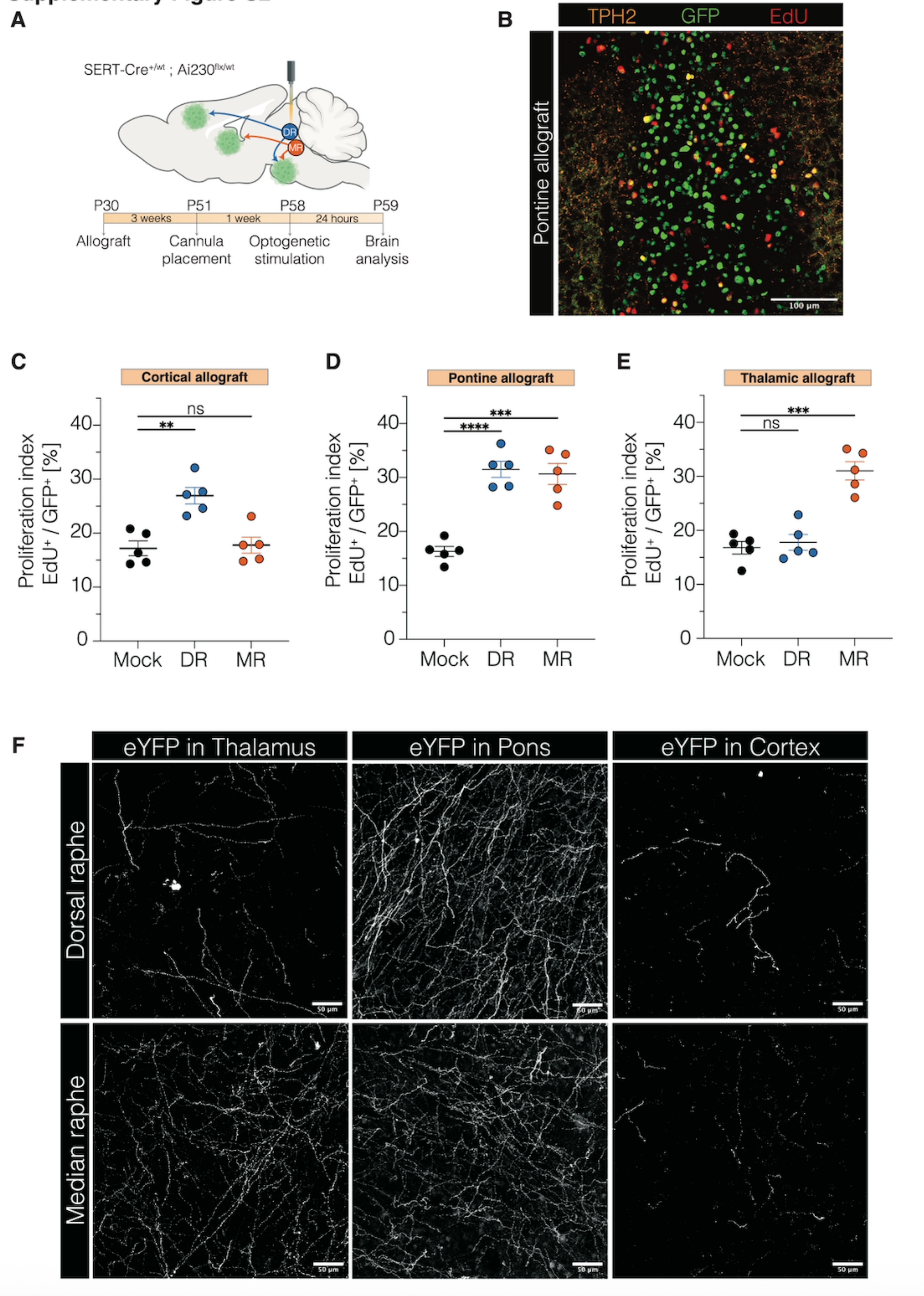

bioRxiv preprint doi: https://doi.org/10.64898/2025.12.10.693579; this version posted December 12, 2025. The copyright holder for this preprint (which was not certified by peer review) is the author/funder, who has granted bioRxiv a license to display the preprint in perpetuity. It is made available under aCC-BY 4.0 International license.

Fig. S2: 5HT neuronal activity promotes glioma proliferation in a circuit-dependent manner across distinct anatomical locations.

- **A.** Schematic of experimental paradigm for optogenetic stimulation of DR^{5HT} and MR^{5HT} neurons in mice bearing H3K27M DMG. Four-week-old SERT-$Cre^{+/wt}$ x $Ai230^{flx/wt}$ mice (P28-30) were allografted with a H3K27M MADR tumor model into neocortex (M2), thalamus, or ventral pons, with optic ferrule placement into the DR or MR three weeks after allografting. Optogenetic stimulation for 30 minutes of the DR or MR was performed four weeks after allografting, followed by perfusion 24 hours after stimulation.
- **B.** Representative confocal image showing GFP-labeled DMG cells in the ventral pons and 5HT projections labeled by TPH2. GFP: green, TPH2: orange, EdU: red, scale bar = 100 µm.
- **C.** Proliferation index (EdU^+/GFP^+) of cortical allografts in mice either stimulated in DR or MR or mock-stimulated ("Mock") (Mock, DR, and MR, n=5 mice/group). Unpaired two-tailed Welch's t-test; $**p < 0.01$, ns: non-significant. Data=mean ± SEM.
- **D.** Proliferation index (EdU^+/GFP^+) of ventral pontine allografts in mice either stimulated in DR or MR or mock-stimulated ("Mock") (Mock, DR, and MR, n=5 mice/group). Unpaired two-tailed Welch's t-test; $****p < 0.0001$, $***p < 0.001$. Data=mean ± SEM.
- **E.** Proliferation index (EdU^+/GFP^+) of thalamic allografts in mice either stimulated in DR or MR or mock-stimulated ("Mock") (Mock, DR, and MR, n=5 mice/group). Unpaired two-tailed Welch's t-test; $***p < 0.001$, ns: non-significant. Data=mean ± SEM.
- **F.** Representative confocal images showing virally labeled 5HT projections from the MR (left images) and DR (right images) in the thalamus, ventral pons, and cortex. eYFP: white, scale bar = 50 µm.

bioRxiv preprint doi: https://doi.org/10.64898/2025.12.10.693579; this version posted December 12, 2025. The copyright holder for this preprint (which was not certified by peer review) is the author/funder, who has granted bioRxiv a license to display the preprint in perpetuity. It is made available under aCC-BY 4.0 International license.

Supplementary Figure S3

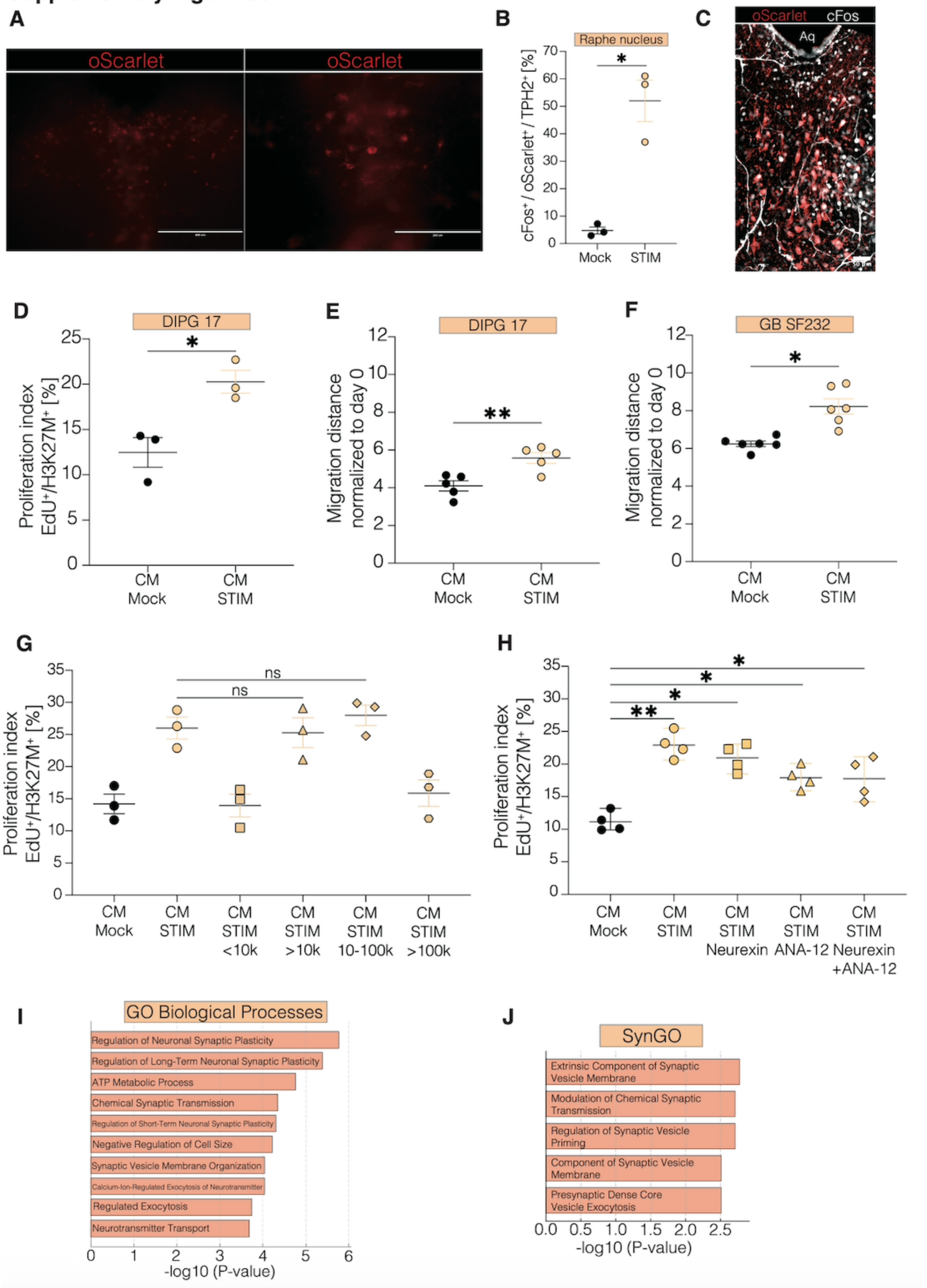

bioRxiv preprint doi: https://doi.org/10.64898/2025.12.10.693579; this version posted December 12, 2025. The copyright holder for this preprint (which was not certified by peer review) is the author/funder, who has granted bioRxiv a license to display the preprint in perpetuity. It is made available under aCC-BY 4.0 International license.

Fig. S3: Conditioned media derived from 5HT neuronal activity in the raphe nucleus promotes glioma cell proliferation and migration.

A. Representative confocal images showing local oScarlet-labeled ChRmine expression in the raphe nucleus in live *ex vivo* active slices. oScarlet: red, scale bar = 200 µm.

B. Quantification of cFos expression following *ex vivo* optogenetic stimulation of 5HT neurons in the RN ("STIM") compared to mock-stimulated controls ("Mock") in active slices of SERT-Cre x Ai230 mice. Unpaired two-tailed Welch's t-test; $^*p < 0.05$. Data are presented as mean ± SEM.

C. Confocal micrograph showing cFos expression in the raphe nucleus fixed active slices. oScarlet: red, cFos: white, scale bar = 50 µm.

D. Quantification of DMG cell proliferation ($EdU^+/H3K27M^+$) when adding CM after *ex vivo* stimulation of RN compared to CM from mock-stimulated slices. Unpaired two-tailed Welch's t-test; $^*p < 0.05$. Data=mean ± SEM; n=three independent experiments, each with three wells per condition; each data point represents the mean of three wells per condition for a given experiment.

E. Quantification of DMG cell migration after 72hours when adding CM after *ex vivo* stimulation of RN compared to CM from mock-stimulated slices. Unpaired two-tailed Welch's t-test; $^{**}p < 0.01$. Data=mean ± SEM; n=five independent experiments, each with three wells per condition; each data point represents the mean of three wells per condition for a given experiment.

F. Quantification of glioblastoma cell migration after 72hours when adding CM after *ex vivo* stimulation of RN compared to CM from mock-stimulated slices. Unpaired two-tailed Welch's t-test; $^*p < 0.05$. Data=mean ± SEM; n=five independent experiments, each with three wells per condition; each data point represents the mean of three wells per condition for a given experiment.

bioRxiv preprint doi: https://doi.org/10.64898/2025.12.10.693579; this version posted December 12, 2025. The copyright holder for this preprint (which was not certified by peer review) is the author/funder, who has granted bioRxiv a license to display the preprint in perpetuity. It is made available under aCC-BY 4.0 International license.

G. Proliferation index ($EdU^+/H3K27M^+$) of a DMG cell line ("SU-DIPG17") after fractionation of the CM by molecular weight. One-way analysis of variance (ANOVA) with Tukey's post hoc analysis; ns: non-significant. Data=mean ± SEM; n=three independent experiments, each with three wells per condition; each data point represents the mean of three wells per condition for a given experiment.

H. Proliferation index ($EdU^+/H3K27M^+$) of a DMG cell line ("SU-DIPG17") after adding a NLGN3-antagonist (Neurexin) and TrkB-inhibitor (ANA-12) to the CM. One-way analysis of variance (ANOVA) with Tukey's post hoc analysis; $^{**}p < 0.01$, $^{*}p < 0.05$, ns: non-significant. Data=mean ± SEM; n=three independent experiments, each with three wells per condition; each data point represents the mean of three wells per condition for a given experiment.

I. Gene ontology terms for biological processes identified in the CM from stimulated active slices, based on the top 50 upregulated proteins.

J. Gene ontology terms using SynGO analysis of the CM from stimulated active slices, based on the top 50 upregulated proteins.

bioRxiv preprint doi: https://doi.org/10.64898/2025.12.10.693579; this version posted December 12, 2025. The copyright holder for this preprint (which was not certified by peer review) is the author/funder, who has granted bioRxiv a license to display the preprint in perpetuity. It is made available under aCC-BY 4.0 International license.

Supplementary Figure S4

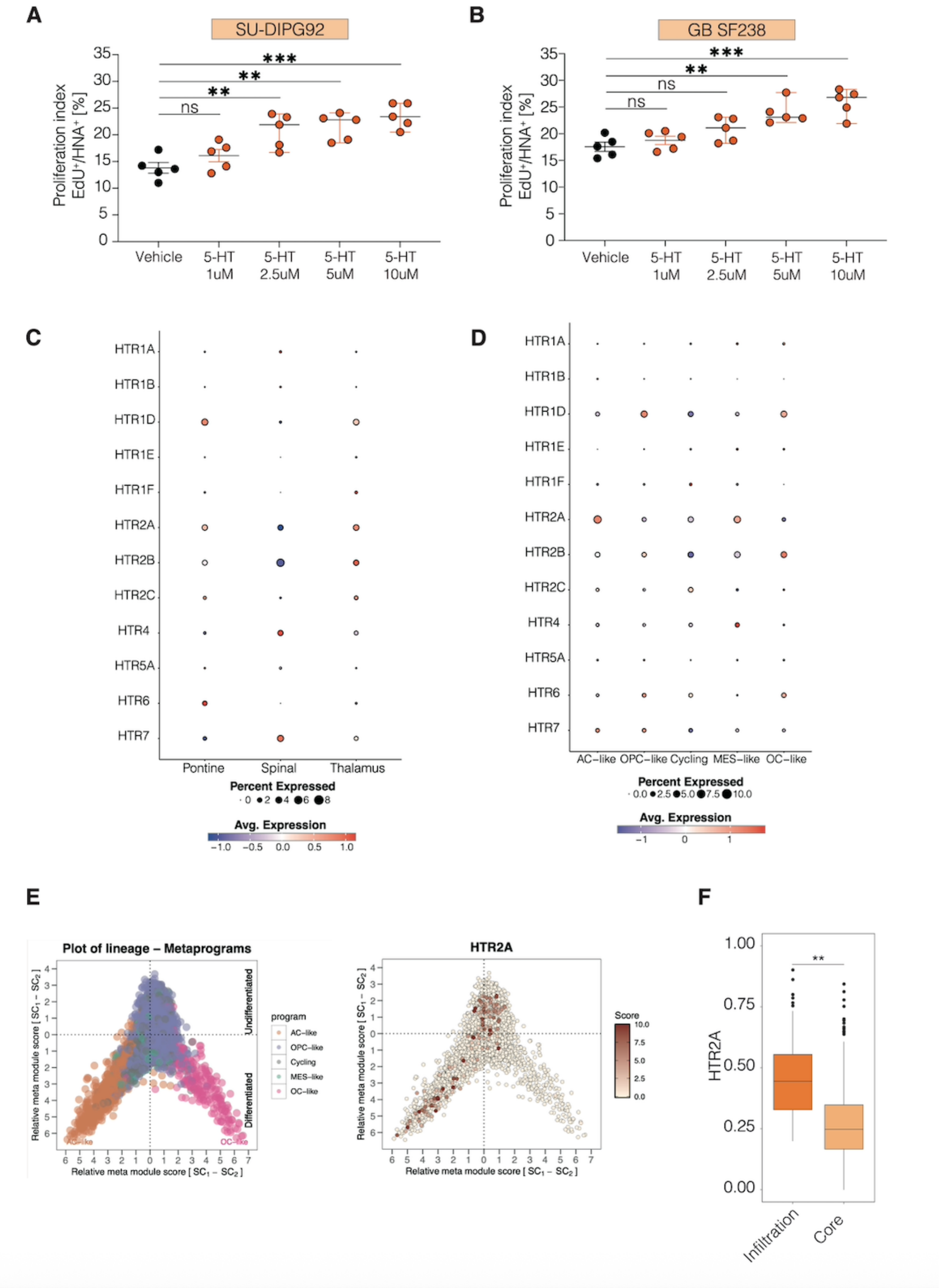

bioRxiv preprint doi: https://doi.org/10.64898/2025.12.10.693579; this version posted December 12, 2025. The copyright holder for this preprint (which was not certified by peer review) is the author/funder, who has granted bioRxiv a license to display the preprint in perpetuity. It is made available under aCC-BY 4.0 International license.

Fig. S4: HTR2A is the most upregulated serotonergic receptor in both diffuse midline glioma and glioblastoma.

- **A.** Proliferation index ($EdU^+/DAPI^+$) of monocultures of a patient-derived DMG line treated with varying doses of 5HT. One-way ANOVA with Tukey's post hoc test; ***p < 0.001, **p < 0.01, *p < 0.05, ns: non-significant. Data are presented as mean ± SEM. n=five to six independent experiments, each with three wells per condition; each data point represents the mean of three wells per condition for a given experiment.
- **B.** Proliferation index ($EdU^+/DAPI^+$) of monocultures of a patient-derived adult glioblastoma line treated with varying doses of 5HT. One-way ANOVA with Tukey's post hoc test; ***p < 0.001, **p < 0.01, *p < 0.05, ns: non-significant. Data are presented as mean ± SEM. n=five to six independent experiments, each with three wells per condition; each data point represents the mean of three wells per condition for a given experiment.
- **C.** Illustration of serotonergic receptor gene expression in human DMG samples, showing the number of glioma cells expressing the gene and the average expression level across ventral pons, thalamus, and spinal cord.
- **D.** Illustration of serotonergic receptor gene expression in human DMG samples, showing the number of glioma cells expressing the gene and the average expression level across different glioma cell states.
- **E.** HTR2A expression level in malignant H3K27M+ malignant single cells projected on the metaprograms (x axis) and stemness (undifferentiated to differentiated; y axis) scores.
- **F.** Comparison of HTR2A expression between the tumor core and infiltration zone based on spatial transcriptomic data from human glioblastoma samples. Unpaired two-tailed Welch's t-test; **p < 0.01.

bioRxiv preprint doi: https://doi.org/10.64898/2025.12.10.693579; this version posted December 12, 2025. The copyright holder for this preprint (which was not certified by peer review) is the author/funder, who has granted bioRxiv a license to display the preprint in perpetuity. It is made available under aCC-BY 4.0 International license.

Supplementary Figure S5

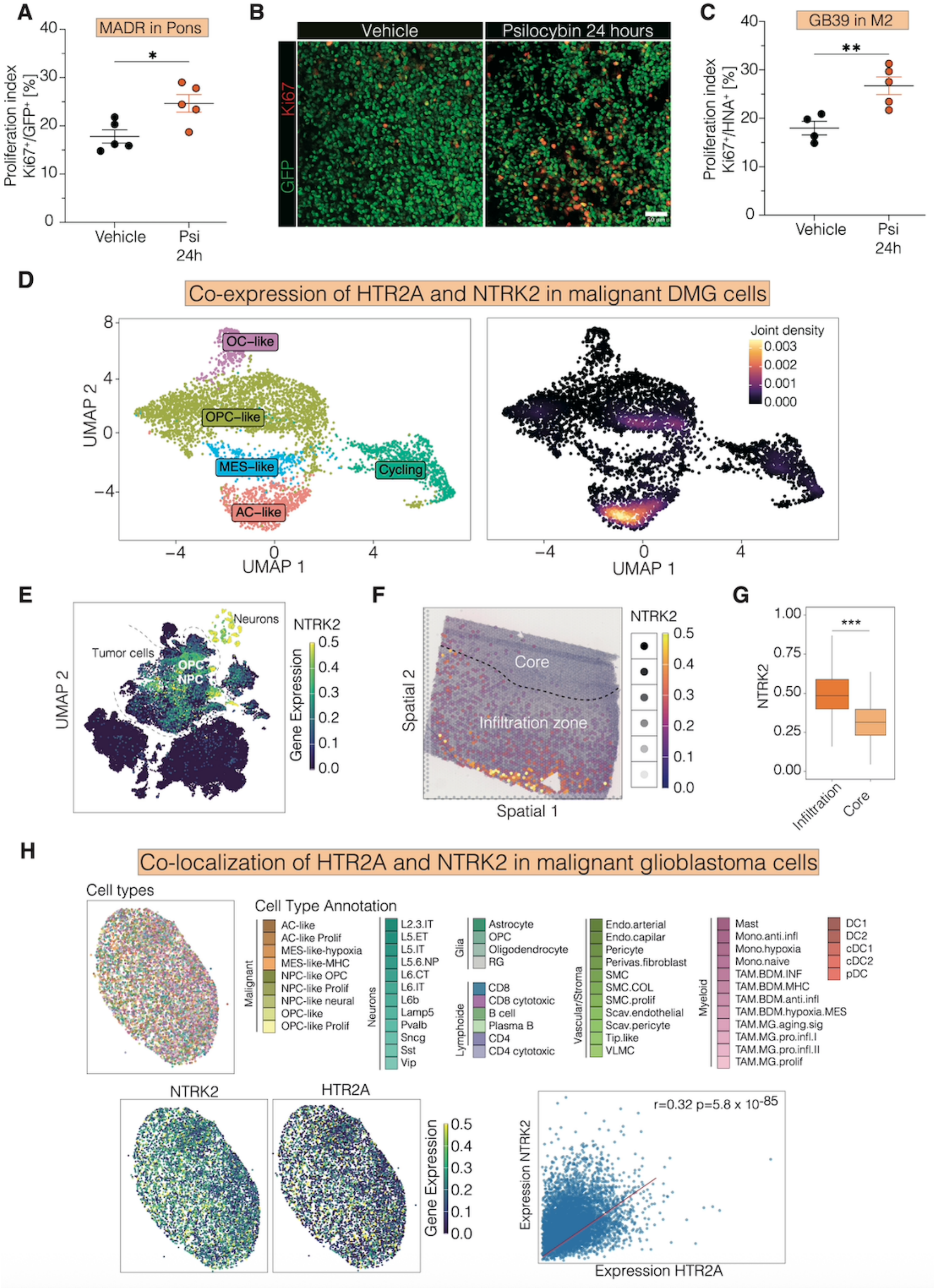

bioRxiv preprint doi: https://doi.org/10.64898/2025.12.10.693579; this version posted December 12, 2025. The copyright holder for this preprint (which was not certified by peer review) is the author/funder, who has granted bioRxiv a license to display the preprint in perpetuity. It is made available under aCC-BY 4.0 International license.

Fig. S5: Psilocybin effects on glioma cell proliferation and co-expression of HTR2A and NTRK2.

A. Proliferation index ($Ki67^{+}/GFP^{+}$) of ventral pontine allografts (MADR) in mice treated with psilocybin ('Psi') or vehicle control ('Vehicle') (n = 5 mice/group). Unpaired two-tailed Welch's t-test; $^{*}p < 0.05$. Data are presented as mean ± SEM.

B. Representative confocal micrograph showing proliferation rates in ventral pontine allografts following treatment with psilocybin or vehicle control. Ki67: red, GFP: green, scale bar = 50 μm.

C. Proliferation index ($Ki67^{+}/HNA^{+}$) of cortical xenografts (GB39) in mice treated with psilocybin ('Psi') or vehicle control ('Vehicle') ('Psi', n = 5 mice; 'Vehicle', n = 4 mice). Unpaired two-tailed Welch's t-test; $^{**}p < 0.01$. Data are presented as mean ± SEM.

D. Single-cell analysis of human DMG samples showing co-expression of HTR2A and NTRK2 within the same glioma cells, clustered by malignant metaprograms.

E. UMAP plot illustrating NTRK2 expression across different cell types and cell states based on single-cell sequencing datasets from human glioblastoma samples.

F. Analysis of spatial NTRK2 expression in human glioblastoma samples,

G. Expression levels of NTRK2 compared between the tumor infiltration zone and core.

H. Spatial transcriptomic analysis of human glioblastoma samples showing cell type–dependent expression of HTR2A and NTRK2, and their spatial co-localization and correlation within the tumor microenvironment.

bioRxiv preprint doi: https://doi.org/10.64898/2025.12.10.693579; this version posted December 12, 2025. The copyright holder for this preprint (which was not certified by peer review) is the author/funder, who has granted bioRxiv a license to display the preprint in perpetuity. It is made available under aCC-BY 4.0 International license.

Supplementary Figure S6

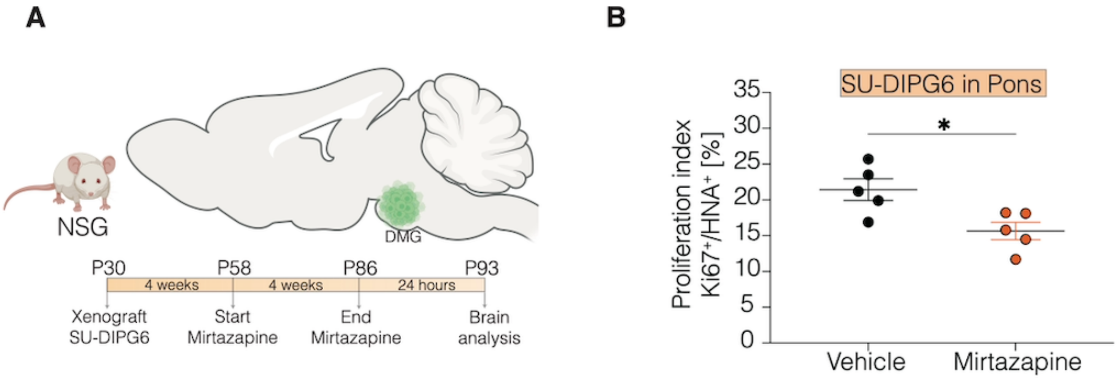

bioRxiv preprint doi: https://doi.org/10.64898/2025.12.10.693579; this version posted December 12, 2025. The copyright holder for this preprint (which was not certified by peer review) is the author/funder, who has granted bioRxiv a license to display the preprint in perpetuity. It is made available under aCC-BY 4.0 International license.

Fig. S6: Mirtazapine reduces glioma cell proliferation.

A. Schematic of the experimental paradigm for xenografting and chronic mirtazapine treatment. Four-week-old NSG mice (P28–30) were xenografted in the ventral pons with patient-derived DMG cells (SU-DIPG6). Daily intraperitoneal mirtazapine treatment (10 mg/kg body weight) was initiated four weeks post-xenograft and continued for four consecutive weeks.

B. Proliferation index ($Ki67^+/HNA^+$) of ventral pontine xenografts in mice treated with the mirtazapine or vehicle control ($n = 5$ mice/group). Unpaired two-tailed Welch's *t*-test; $^*p < 0.05$. Data are presented as mean ± SEM.
